# Male Reproductive Health - study of a sperm donor population

**DOI:** 10.5935/1518-0557.20210064

**Published:** 2022

**Authors:** Ana Catarina Silva Fonseca, Márcia Barreiro, António Tomé, Emídio Vale-Fernandes

**Affiliations:** 1 USF Serpa Pinto, ACeS Grande Porto V - Porto Ocidental, Porto, Portugal; 2 Instituto de Ciências Biomédicas Abel Salazar, Universidade do Porto, Porto, Portugal; 3 Centro de Procriação Medicamente Assistida / Banco Público de Gâmetas, Centro Materno-Infantil do Norte Dr. Albino Aroso (CMIN), Centro Hospitalar Universitário do Porto (CHUP), Porto, Portugal; 4 Departamento da Mulher e da Medicina Reprodutiva, Centro Materno-Infantil do Norte Dr. Albino Aroso (CMIN), Centro Hospitalar Universitário do Porto (CHUP), Porto, Portugal; 5 Unidade Multidisciplinar de Investigação Biomédica (UMIB), Instituto de Ciências Biomédicas Abel Salazar (ICBAS), Universidade do Porto, Porto, Portugal

**Keywords:** male fertility, sperm donors, medically assisted reproduction, public gamete bank

## Abstract

**Objective:**

Every individual has the right to a safe and satisfactory sex and reproductive life. Therefore, several countries have made efforts to make Reproductive Health resources available to their populations. However, few results have been published regarding the policies implemented in Portugal. This study looked into the reproductive health status of the Portuguese male population.

**Methods:**

Sperm donor candidates from the Portuguese Public Gamete Bank registered between 2011 and 2018 were included in the study. Spermogram findings were evaluated with respect to sociodemographic and risk factors.

**Results:**

This is the first study performed in this population. We found that sperm quality has decreased throughout the last nine years, and that spermatozoa progressive motility is inversely correlated with the body mass index. An association between drug use and decreased sperm pH was also found.

**Conclusions:**

Changes in sperm quality have important consequences in male fertility. Most of the identified causes of decreased sperm quality are modifiable factors and should therefore be addressed since an early age.

## INTRODUCTION

The definition of Sexual and Reproductive Health was introduced in 1994 during the International Conference on Population and Development. It includes the right to a safe and satisfactory sex life as well as the right to decide if, when and how often a person wants to have children (DGS, 2008; Fathalla & Fathalla, 2017).

Infertility, defined as the inability to conceive after 12 months of unprotected and frequent intercourse, affects approximately 8-12% of couples worldwide (Vander Borght & Wyns, 2018). According to Portuguese law, medically assisted reproduction can be offered to heterosexual couples with infertility as well as couples of two women and single women diagnosed or not with infertility (*Lei No17/2016 - Diário Da República No116/2016, Série I de 2016/06/20*, n.d., 1). In many such cases sperm donors are needed.

Sexual and Reproductive Health is influenced by several factors, including behavior, economy and global health. A careful analysis of samples from healthy gamete donors might help understand the effect of risk factors on fertility. Regarding male fertility, smoking has been associated with decreased sperm quality and quantity, decreased motility, and abnormal sperm cell morphology (Durairajanayagam, 2018). Drinking alcohol has been associated with decreased semen volume, teratozoospermia and suppression of the hypothalamic-pituitary-gonadal axis, leading to reduced spermatogenesis and lower levels of testosterone (La Nasa, 2018). Negative correlations between increased paternal age and several spermogram parameters have also been described, affecting factors such as total number of spermatozoa, sperm motility, concentration, volume and morphology (Durairajanayagam, 2018). Other factors implicated in decreased male fertility include consumption of processed meat, fat dairy and sugary drinks, drugs (including cocaine, marijuana and steroids) as well as having a higher body mass index (Camlin *et al*., 2016; Durairajanayagam, 2018; La Nasa, 2018).

The first Portuguese Public Gamete Bank (PGB) was created in 2011 at *Centro Hospitalar Universitário do Porto* (CHUP), and subsequently, a national network of affiliated public centers was developed. The PGB and its affiliated centers are responsible for the entire process, starting from donor recruitment to cryopreservation, while storage and distribution are the responsibility of the PGB at CHUP (*Despacho n.o 3219/2011 de 17 de Fevereiro - Diário Da República - 2.a Série, No 34, Pág. 8375*, n.d.; "Despacho n.o 679/2017 de 11 de Janeiro- Diário da República n.o 8/2017, Série II de 2017-01-11, páginas 1098 - 1099" n.d.). The PGB in Portugal accepts male donors aged between 18 and 40 years. Donors can perform no more than seven sperm donations, and the sperm each donor gives away may be used to originate newborns in no more than eight different families. Donations are voluntary and free (Banco Público de Gâmetas" n.d.; *Lei No32/2006 - Diário Da República No143/2006, Série I de 2006/07/26*, n.d.).

The main aim of this study was to evaluate the reproductive health status of the Portuguese male population and to identify the main risk factors that affect male fertility. All potential male gamete donors evaluated at the PGB at CHUP were included and treated as a sample of the nation's population.

## MATERIAL AND METHODS

This single-center retrospective cohort study included sperm donors registered with PGB between May 2011 and December 2018. The CHUP Ethics Review Committee approved the study design.

The information collected from the PGB database included donor age, marital status, occupation, number of children, anthropometric data, phenotype, current and past medical history, family history, occupational exposure, sperm evaluation and serological assessment. All semen samples were collected by masturbation at PGB after a recommended period of two to three days of sexual abstinence. The data were anonymized with the aid of a cross tabulation tool and each participant was assigned a codename.

Until December 2018, a total of 267 individuals were registered with PGB. Twenty-one were excluded from statistical analysis because they did not have sperm values (excluded *a priori* due to poor clinical history or withdrawal). Twenty individuals registered with PGB affiliated centers were also excluded to minimize the biases associated with the way the interviews were conducted. This resulted in a population of 226 individuals.

In terms of alcohol drinking, the possible answers recorded in the PGB database were "Yes", "No", "Yes, socially" and "Yes, during meals". To ensure homogeneity, the answers were divided into "Yes" or "No", depending on whether the participants drank alcohol ("Yes", "Yes socially" and "Yes at meals") or not, respectively. Similarly, the possible answers for smoking were "Yes", "No", "Yes, socially" and "I used to smoke". Therefore, "Yes" and "Yes, socially" were grouped into "Yes", while "No" included "No" and "I used to smoke". Drug use was the parameter with the highest variability in the BPG database, with several possible answers ("Yes", "No", "Yes - marijuana occasionally", "Yes - socially", "Yes - hashish", "Not since three years ago", "Not since one year ago (hashish)" and "In the past"). To standardize the answers, drug use was also sorted into "Yes" or "No" according to whether there was any drug use at the moment of sperm donation.

The data were analyzed on statistical package IBM SPSS Statistics 25. The data are expressed as mean ± standard error of the mean (±SEM) unless otherwise indicated. A significance level of 0.05 was considered statistically relevant. Significance levels are presented as follows: * for *p*<0.05 and ** for *p*<0.01.

The T-test for independent samples was used to compare data sets obtained from independent groups of individuals (such as age, body mass index --BMI-- and risk factors) with sperm parameters. Subsequently, ANOVA was used to assess the differences and interactions between sociodemographic and risk factors and sperm parameters.

The evolution of the various sperm parameters over the years was analyzed using ANOVA. The Tukey Post Hoc test was performed to determine between which years there were significant differences in averages.

Mean values and error bars representing the associated standard error were plotted in graphs, unless otherwise indicated.

## RESULTS

The population featured in this study included sperm donors aged between 18 and 40 years, which comprises the age range to which donors must belong in order to donate gametes in Portugal. Donors were aged 27 years on average (Table 1), but most were aged 30 years and younger. The BMI of the study population followed a normal distribution, and the average BMI was 23.7 kg/m^2^. Regarding marital status, most donors were single (77.9%), and about 55% were students attending high school, vocational school or university (Table 1).

**Table 1.** Sociodemographic characteristics of the study population.

Mean age (± SEM), years	27±0.35
Mean BMI (± SEM), Kg/m^2^	23.7±0.19
Marital status, n (%)	
Single	176 (77.9)
Married	23 (10.2)
Other*	27 (11.9)
Professional status, n (%)**	
Student	124 (54.9)
Employed	79 (35)
Unemployed	14 (6.2)
Risk factors, n (%)	
Alcohol	127 (56.2)
Smoking	45 (19.9)
Drugs	20 (8.8)

* Other: living together for over 2 years, divorced, undefined

** 9 missing values

Alcohol, tobacco and drug use were analyzed as risk factors for reduced male fertility (Table 1). More than half (56.2%) of the population reported alcohol drinking, almost a fifth (19.9%) smoked some type of tobacco at the time of donation, and less than a tenth (9%) admitted to using drugs at the time of donation (Table 1).

The subjects in the sample had at least one spermogram done to assess their eligibility as sperm donors. Only the first spermogram was used in this study (main spermogram). The average values for each spermogram parameter (volume, pH, progressive motility, concentration, normal morphology and leukocyte count) are shown in Table 2. About two thirds (66.1%) of the 226 individuals were normozoospermic according to the World Health Organization (WHO) criteria (no sperm parameter outside reference values). While assessing the various sperm parameters, the following diagnoses were identified: 8.4% of individuals with oligozoospermia (n=19), 9.7% with asthenozoospermia (n=22), 23.9% with teratozoospermia (n=54), 3.1% with oligoasthenoteratozoospermia (n=7), 5.3% with oligoteratozoospermia (n=12), and 4.9% with asthenoteratozoospermia (n=11).

**Table 2.** Main spermogram evaluation.

Spermogram parameters	Mean ± SEM	Reference values WHO*	Candidates with abnormal values, % (n)
Volume (mL)	3.1±0.11	≥1.5	11.9 (27)
pH^a^	8.0±0.02	≥7.2	0.4 (1)
Progressive Motility (%)	54.1±1.06	≥32	9.7 (22)
Concentration (*106/mL)	81.5±4.28	≥15	8.4 (19)
Normal Morphology (%)^b^	7.4±0.25	≥4	23.9 (54)
Leukocytes (*106/mL)	0.5±0.16	≤1	5.8 (13)

a 4 missing values

b 1 missing value

* World Health Organization (2010); (*Cooper* et al., 2010)

**Figure 1:**
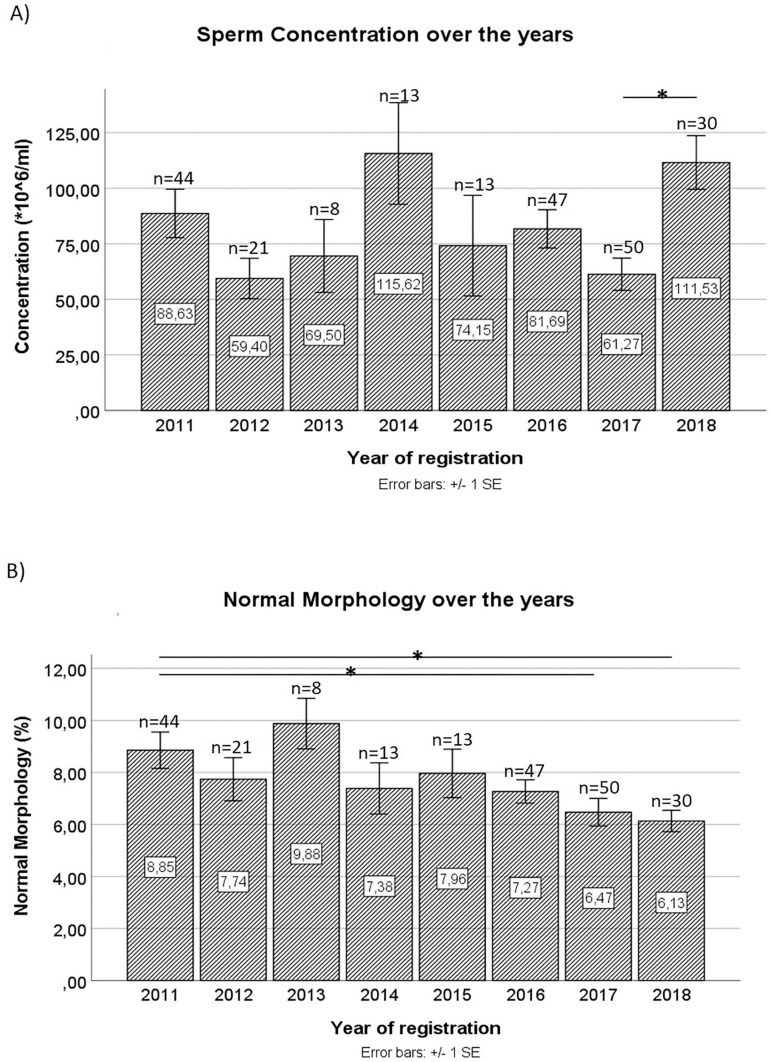
Sperm concentration (A) and Normal Morphology (B) over the years; *p<0.05.

The evolution of each of the spermogram parameters over the years was also analyzed. A statistically significant decrease was found in the average value of sperm concentration in the ejaculate in 2017 (61.27±7.27) compared to 2018 (111.53±12.11, *p*=0.014) (Figure 1A). A progressive decrease in the percentage of spermatozoa with normal morphology in the ejaculate was also observed from 2011 to 2018. In fact, there was a statistically significant difference between the mean values in 2011 (8.85±0.70) and 2017 (6.47±0.53) and between 2011 and 2018 (6.13±0.41).

**Figure 2:**
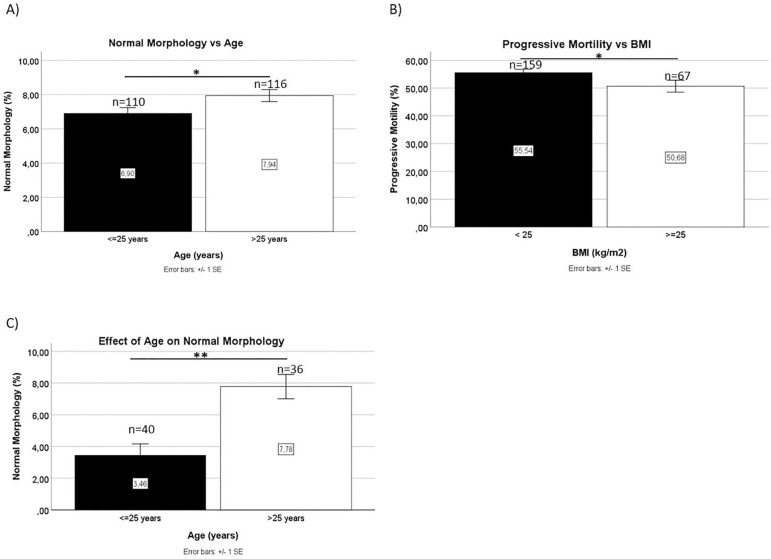
Mean values of Normal Morphology as a function of age (A) and Progressive Motility as a function of BMI (B); effect of Age on Normal Morphology in non-normozoospermic men (C); *p<0.05, **p<0.01.

The relationships between spermogram values and sociodemographic and risk factors were analyzed. Since about half of the individuals in the population were aged 25 years or younger (48.7%), this age was used as a cutoff value for the remaining statistical analyses. The evaluation of various sperm parameters found a statistically significant difference in the percentages of spermatozoa with normal morphology. The group of individuals aged 25 years or under had an average of 6.9% (± 0.34) spermatozoa with normal morphology, while older individuals had on average 7.9% (± 0.35) spermatozoa with normal morphology (Figure 2A). There were no other statistical differences in the remaining spermogram parameters.

The sample was subdivided into two categories according to BMI, thereby dividing donors into groups with subjects with a "Normal BMI" (BMI<25 kg/m^2^) or "Overweight/Obese" (BMI≥25 kg/m^2^). Overweight/obese individuals had lower mean progressive motility (50.7±2.14) than subjects with a normal BMI (55.5±1.20) (Figure 2B).

Risk factors for changes in spermogram parameters were tobacco, alcohol and drug use. In this population, most donor candidates did not use tobacco or drugs. However, more than half of the individuals reported drinking alcohol regularly (during meals or socially). The PGB database does not contain information on the number of grams of alcohol ingested per day, which would allow for a more accurate intake assessment. Regarding tobacco, there was a statistically significant difference in the number of leukocytes present in the ejaculate of smokers versus non-smokers. In this sample, donor candidates who were smokers had an average of 0.1 (± 0.05) million white blood cells per milliliter, compared with 0.6 (± 0.20) million white blood cells per milliliter in non-smokers (data not shown), with an average age of 26.2 and 26.8 years, respectively. However, there were no statistical differences in any of the sperm parameters between individuals with a history of drinking alcohol or using drugs and non-drinkers and non-drug users.

ANOVA was performed to monitor the effect of various sociodemographic (age and BMI) and risk factors (tobacco, alcohol and drug use) as well as their interactions with different spermogram parameters.

Drug use was associated with lower pH levels. Spermograms from non-drug users had a mean pH of 8.09±0.031, while drug users had a pH of 7.86±0.082 on average [F (1, 201) = 6.823, *p*=0.010]. Nevertheless, the pH value remained above the normal value for the population (pH≥7.2) according the WHO criteria.

As for the remaining spermogram parameters, namely Progressive Motility, Concentration and Leukocyte Count, there were no significant interactions between the various factors tested.

Statistical evaluation by ANOVA was repeated using the same criteria, but considering only individuals with at least one altered spermogram parameter and excluding normozoospermic subjects.

Analysis of the percentage of spermatozoa with normal morphology indicated that this parameter was affected by age [F (1, 58) = 7.010, *p*=0.010]. In our study, individuals aged 25 years or younger had on average a percentage of normal-shaped sperm below the value considered normal for the population (3.46±0.707), with a significant difference from individuals aged 25 years or older (7.78±0.767) (Figure 2C). There were no differences between the two groups related to risk factor consumption.

There were no other associations between the various sociodemographic and risk factors and the remaining spermogram parameters.

## DISCUSSION

The success of fertilization, whether natural or through medically assisted reproduction methods, is closely related to male and female gamete characteristics. Therefore, the potential donors registered with PGB were used as a sample of the Portuguese male population in order to study sperm quality and determine the effects of sociodemographic and risk factors on sperm parameters.

In the study population, the main spermatozoa abnormalities found, in decreasing order of prevalence, were teratozoospermia, asthenozoospermia and oligozoospermia. These results were similar to the data published by Ombelet *et al*. (2009), who conducted a study at a fertility clinic in Belgium, where they evaluated the partners of women with chronic anovulation. Studies like these allow the indirect assessment of the prevalence of spermatozoa abnormalities in the general population. This is of major importance, given that almost a quarter of this study's population had teratozoospermia, which compromises the effectiveness of fertilization, both *in vivo* and *in vitro*. Some studies demonstrated a significant positive correlation between the percentage of spermatozoa with normal morphology and the fertilization index, as only some of the spermatozoa with normal shapes are able to undergo the acrosome reaction to consequently fuse with the oocyte (Szczygiel & Kurpisz, 1999).

Our study found that both sperm concentration and normal morphology differed significantly over the years. Although Concentration does not appear to clearly decline over the years, we found a significant decrease in average concentration levels in 2017 when compared to 2018. Also, 2017 was the year with the highest number of sperm donations. This data is very relevant, since it shows that we might be overestimating the actual sperm concentration in our population, given that larger samples yield more representative results regarding the overall population. Therefore, 2017 results might be more significant than the results from other years. However, we found a clear trend towards decreased levels of sperm with normal morphology over the years. Between 2011 and 2017 and 2018, this parameter decreased by 26.89% and 30.73%, respectively. These results are in line with the data reported by van den Hoven *et al*. (2015), who demonstrated that between 1986 and 2011 there was a decrease in the levels of morphologically normal spermatozoa, in a study carried out in the Netherlands. The authors reported a decrease from 30-80% to 0-10% of sperm with normal morphology at the end of the study. Splingart *et al*. (2012) published similar results in a study conducted on a population of sperm donors in France between 1976 and 2009. Since our study included data from a later period than other studies published in the literature, it may suggest new evidence on the continued decline in the percentage of spermatozoa with normal morphology in the ejaculate. However, it should be noted that such prior studies were carried out with different populations. Nonetheless, there are no studies based on the Portuguese population, which would be important to both confirm that this data is representative of the population, and to determine whether the decreases seen in some spermogram parameters account for an older trend.

The effect of maternal age on successful fertilization and reproduction is widely recognized. However, there is no definition for "advanced paternal age". Stone *et al*. (2013) studied the effect of male age on spermogram parameters and concluded that individuals aged up to 34 years had no variation in sperm parameters. On the other hand, from the age of 34 onwards, there was a decrease in the total number of spermatozoa, a decrease in concentration and levels of sperm with normal morphology starting at 40 years of age, and a decrease in ejaculate volume starting at the age of 45. A literature review described a decrease in levels of sperm with normal morphology between the ages of 30 and 50 years (Kidd *et al*., 2001). In Brazil, Oliveira *et al*. (2014) correlated aging with decreasing levels of sperm with normal morphology, progressive motility and spermatozoa vitality, as well as with increased sperm DNA fragmentation. However, we found that the youngest individuals in our population, the group aged less than 25 years, had fewer spermatozoa with normal morphology than the group aged between 26 to 40 years, with no additional variations in the remaining spermogram parameters. However, normal morphology values in both groups indicated normozoospermia.

On the other hand, when evaluating exclusively donor candidates that had at least one altered spermogram parameter, we found that younger age correlated with a decline in the levels of sperm with normal morphology, leading to a diagnosis of teratozoospermia. Nonetheless, it should be noted that these results were not controlled for risk factors or the number of days of abstinence. In fact, when controlled for risk factors, this variation was no longer observed. Moreover, the number of days of abstinence is an important confounding factor (Auger *et al*., 1995). Although present in the PGB database, this information is not systematically populated; therefore, we were unable to use it to control the remaining parameters. Also, the literature uses the age of 35 as a cutoff, while in this study we used the age of 25, since Portuguese donors are aged between 18 and 40 years. The authors of the present study understand that this is a rather counter-intuitive result. However, this is the first study carried out in Portugal on the subject and the authors believe these findings warrant further investigation. Sexual and reproductive health measures, especially in what concerns male populations, is sometimes assigned a lower level of priority within the realm of health policies. If future studies corroborate our findings, we might begin to see the implementation of new reproductive health policies designed to improve birth rates.

A decrease in progressive motility was also observed as the BMI increased. This finding is consistent with recent studies that demonstrated a negative correlation between these two parameters. In fact, Belan *et al*. (2019) demonstrated not only a decrease in progressive motility, but also in the total number of spermatozoa, while Bieniek *et al*. (2016) described a negative correlation between the BMI and sperm volume, concentration, motility and morphology. Campbell *et al*. (2015) had already identified a negative correlation between the BMI and progressive motility. Excess weight/obesity is particularly relevant. According to data from the *Direção-Geral da Saúde* (DGS), the Portuguese population has seen increases in the number of overweight/obese individuals between 1960 and 1990. This data was taken from the only study about the progress of obesity in Portugal, carried out in military inspections and targeting 20-year-old males (DGS, 2017). This reinforces the importance of implementing measures to reduce excess weight/obesity, not only due to the associated metabolic and cardiovascular risk, but also on account of the negative impact such factors may have on male reproductive health.

According to the literature, smoking increases the levels of reactive oxygen species, leading to lower levels of semen volume, sperm progressive motility, sperm with normal morphology and sperm viability, and increased levels of immobile spermatozoa and leukocytes in the ejaculate (Sharma *et al*., 2016; Zhang *et al*., 2013). The American Society for Reproductive Medicine issued an opinion stating that the effect of tobacco on male fertility was difficult to discern, because despite the decrease in several parameters, they remain within normal limits (Practice Committee of the American Society for Reproductive Medicine, 2018). In the present study there was an inverse relationship between tobacco consumption and the presence of leukocytes in the ejaculate, with no significant variations in other parameters. However, it is important to note that the studies in the literature evaluating individuals who sought medically assisted reproduction clinics for infertility do not specify whether the patients suffered from male of female infertility, thereby apparently introducing bias selection. In our study, the use of candidates for male gamete donors as a sample of the population allows the reduction of selection bias, since it should comprise a healthy sample of the population. Nevertheless, increased leukocyte counts should be expected in smokers. This apparent inconsistency can be explained by the great variability in leukocyte counts in non-smokers, which might be related with individual variability itself or even with uncontrolled systemic factors, such as presence of infection. Infectious diseases were not controlled for in statistical analysis, since donor candidates excluded for not presenting adequate main spermograms were not tested for infectious diseases. On the other hand, smokers occurred in smaller numbers and had less variation, which may have contributed to the identification of statistically significant differences.

An important confounding factor that was not possible to control in this study was the amount of alcohol consumed per day, as well as the length of time for which participants had been drinking alcohol. Alcohol intake was only qualitatively evaluated. In future studies it might be important to control for this variable by determining the daily or weekly intake of alcohol. The same applies to drug use.

A number of other complex variables interact with the organisms of human beings. Therefore, we looked into the interactions between various sociodemographic and risk factors and sperm quality. We found a significant decrease in ejaculate pH associated with drug use, when controlled for the remaining factors, although mean pH levels resided ​​above the lower reference limit. This is relevant because semen pH is important for sperm viability and mobility, as in more acidic environments there is a decrease in both parameters (Zhou *et al*., 2015). However, there were no other interactions, most likely due to the small number of individuals present in each subcategory.

Other biases include geographical differences and selection bias. Only potential male gamete donors were included. However, the ideal study should include a random sample of the population. Although gamete donation is a free, voluntary and unpaid process, it would also be interesting to understand what motivated these men to donate sperm. Nonetheless, Portuguese law provides that donors de "reimbursed for expenses incurred or losses directly and immediately resulting from their donations up to a tenth of the current social support index" (*Despacho n.o 3192/2017 de 11 de Abril - Diário Da República n.o 75/2017, Série II de 2017-04-17, Páginas 7192 - 7193*, n.d.). There is only one Portuguese study designed to better understand gamete donor payments by analyzing the views of donors and recipients (Samorinha *et al*., 2020).

In terms of selection bias, students and individuals aged less than 30 years accounted for more than half of our population. The changes verified in this study will have repercussions not only in the present, but also in the future, so it is important to anticipate and prevent the effects on the reproductive health of these young individuals. On the other hand, other variables not considered in our analyses such as ejaculate volume might affect the obtained results. Two thirds of the ejaculate volume comprises seminal vesicle secretions, almost a third is prostate secretions, and about 10% comes from the testis and epididymis.

Several factors may affect ejaculate volume, such as short abstinence period (in the first four days of abstinence there is a daily increase of 11.9% in ejaculate volume), incomplete harvest, and retrograde ejaculation, among others (Roberts & Jarvi, 2009). Finally, the number of days of abstinence might be a confounding factor, since longer abstinence periods have been associated with greater ejaculate volume, sperm concentration and decreased motility (Auger *et al*., 1995).

This was the first Portuguese study to evaluate male reproductive health in gamete donor candidates. It allowed the identification of some interesting results that require further analysis, in addition to some weaknesses in the PGB database that might be improved.
